# Choroidal Thickness in Chinese Children Aged 8 to 11 Years with Mild and Moderate Myopia

**DOI:** 10.1155/2018/7270127

**Published:** 2018-05-31

**Authors:** Ya Qi, Li Li, Fengju Zhang

**Affiliations:** ^1^Beijing Tongren Eye Center, Beijing Tongren Hospital, Beijing Ophthalmology and Visual Science Key Lab, Capital Medical University, Beijing, China; ^2^Department of Ophthalmology, Beijing Children's Hospital, National Key Discipline of Pediatrics, National Children's Health Center, Ministry of Education, Capital Medical University, Beijing, China

## Abstract

**Purpose:**

To investigate macular choroidal thickness (CT), topographical variation, and associations between subfoveal choroidal thickness (SFCT) and age, gender, spherical equivalent (SE), and axial length (AL) in Chinese healthy mild and moderate myopia children aged 8 to 11 years.

**Methods:**

One hundred twenty eyes from 120 healthy children were studied. Children were divided into mild and moderate myopia groups. AL and CT were evaluated. CTs were measured at the fovea, and 1 mm, 2 mm, and 3 mm nasal, temporal, superior, and inferior to the fovea.

**Results:**

SFCT was 252.80 ± 46.95 *µ*m in the whole population. AL was shorter in the mild myopia group (24.18 ± 0.69 mm) than in the moderate myopia group (24.97 ± 0.68 mm, *P* < 0.001), and SFCT was thicker in the mild myopia group (262.00 ± 40.57 *µ*m) than in the moderate myopia group (236.00 ± 55.08 *µ*m, *P*=0.005). The topographical variation was similar in refraction groups. CTs nasal to the fovea thinned gradually and were all significantly thinner than SFCT. CTs in the other three directions gradually thickened and peaked at locations of 2 mm to the fovea. Then, CTs thinned at 3 mm to the fovea. The thickest choroid is located temporal to the fovea. There were significant negative correlations between AL and SFCT in the mild myopia group and the whole population. No other correlations were found.

**Conclusions:**

The topographical variations of choroidal thickness were similar in mild and moderate myopia groups with the thickest locations temporal to the fovea. SFCT was relatively stable in children in narrow range of age and refractive error.

## 1. Introduction

The choroid has several functions such as supplying the outer retina and regulating retina temperature. The changes in choroidal thickness move the retina toward the plane of focus and are correlated with the growth of the sclera and the eye [1]. Use of new optical coherence tomography (OCT) modalities, such as enhanced depth imaging, has led to increased visualization of the choroidal anatomy and made assessment of choroidal thickness possible. Many studies involved in choroidal thickness measurement and relationships between choroidal thickness and systemic and ocular parameters such as refractive error and axial length have been carried out in peoples of different countries, ages, and ethnicities [2–8]. But most subjects in the studies were selected in a wide range of age and refractive error, even in the researches aimed at children. The choroidal thickness obtained in the studies might be general if the age range of the subjects was from 3 to 17 years [9], from 6 to 19 years [10], and the refractive error range was from −9.00 diopters (D) to +5.25 D [11], from −11.38 D to +8.38 D [10]. The correlations between choroidal thickness and other ocular biological parameters are significant in wide range of age and refractive error. But conflicting results might conclude in the studies of narrow age and refractive error range. The correlation of the choroidal thickness with refractive error was not significant when the spherical equivalent was −0.43 ± 1.42 D (range, −4.00 D to +2.25 D) [8]. In the multipoint measurement of choroidal thickness, none of the choroidal thickness displayed a significant correlation with age when the age of study population was 11.5 ± 1.7 years (range, 7 to 15 years) [12]. In the study of high myopia, axial length was not related statistically to choroidal thickness in the control group when the refractive error was −0.3 ± 1.8 D (range, −6 D to +6 D) [6].

In this study, we aimed at investigating macular choroidal thickness in a narrow range of age and refractive error. It is well known that the prevalence of myopia in the global teenagers is increasing year by year [13], especially in some countries of East Asia and Southeast Asia [14]. It has been reported that, in China, the prevalence of myopia was 3.9% in first-grade primary school students and it reached to 67.3% in junior middle school first-grade students [15]. Myopia progresses and becomes common in children at the age of 8–11. This study explored the characteristics of choroidal thickness in children with mild and moderate myopia of this age group.

## 2. Materials and Methods

### 2.1. Study Population

A total of 120 healthy children (120 eyes) were enrolled from January 2017 to June, at the Department of Ophthalmology, Beijing Children's Hospital, Capital Medical University, Beijing, China. The inclusion criteria were 8∼11 years of age, best corrected visual acuity ≥1.0, refractive error (spherical equivalent, SE) ranging between −0.50 D and −5.75 D with astigmatism ≤1.5 D, and normal intraocular pressure (IOP ≤ 21 mmHg), without retinal or choroidal abnormalities. The exclusion criteria were a history of retinal and choroidal diseases, a history of glaucoma, a history of prematurity, a history of eye surgery, strabismus, or systemic diseases.

The enrolled children were allocated into two groups according to the SE. Mild myopia was defined as −0.50 D ≥ SE > 3.00 D and moderate myopia as −3.00 D ≥ SE > −6.00 D.

This study was approved by our Institutional Review Board, and all procedures conformed to the tenets of the Declaration of Helsinki.

### 2.2. Ocular Examination

All examinations were obtained between 9 a.m. and 11 a.m. to avoid diurnal variations [16, 17].

All participants underwent a complete ophthalmic examination, including visual acuity, evaluation of anterior segment with slit lamp biomicroscopy, fundus examination with direct ophthalmoscopy, assessment of ocular motility and alignment, objective cycloplegic refraction, IOP, and axial length (AL) measurements. The IOP was determined by noncontact tonometry (CT-1; Topcon, Tokyo, Japan). Refractive error was measured 30 minutes after administration of a fourth drop of a cycloplegic agent (4 drops of 0.5% tropicamide, separated by 5 minutes), using an autorefractor (KR-8900; Topcon, Tokyo, Japan). Axial length was measured using IOLMaster 500 (Carl Zeiss Meditec, Jena, Germany).

### 2.3. OCT Measurements

Choroidal thickness (CT) was visualized by spectral-domain OCT (Spectralis, Heidelberg Engineering, Heidelberg, Germany) with the enhanced depth imaging system and scanned using 9 mm line scan and eye-tracking systems. The wavelength of the OCT instrument was 870 nm, and the scanning speed was 40,000 nm/second. The axial resolution was 5 *µ*m.

Choroidal thickness was determined as the distance from the outer border of the retinal pigment epithelium (RPE) to the inner sclera border and was measured manually by one doctor who was not aware of the participant characteristics at the time of the measurement, using the software provided by the OCT system. Only right eyes were included.

**Figure 1:**
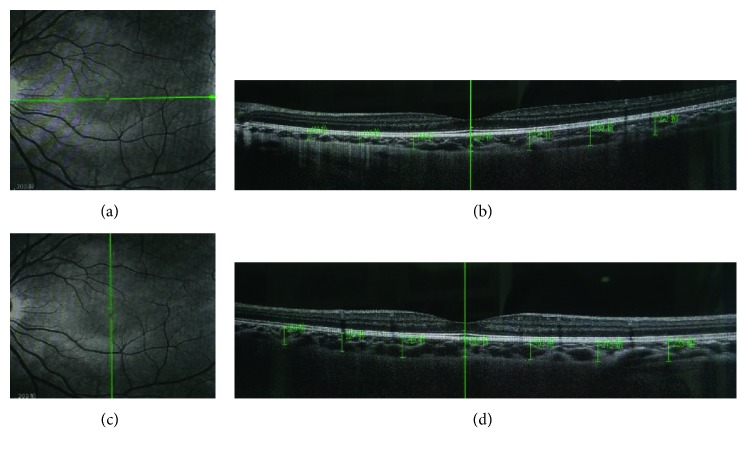
Choroidal scans and thickness measurements (a, b) horizontally and (c, d) vertically on SD-OCT.

A total of 14 locations were measured in each participant, including the fovea (horizontal and vertical), 1 mm, 2 mm, and 3 mm inferior (I), superior (S), nasal (N), and temporal (T) to the fovea (Figure 1). The subfoveal choroidal thickness (SFCT) was calculated as the mean of the horizontal and vertical measured subfoveal choroidal thickness. The intraobserver reliability of choroidal thickness measurements had been evaluated by randomly selected images of 20 eyes to be reassessed by the same doctor.

### 2.4. Statistical Analyses

Measurements used for data analysis were obtained only from the right eye of each participant. IBM SPSS statistics version 21.0 (IBM Co., Armonk, NY, USA) was used for all the statistical analyses. The distribution of each parameter was assessed using the Kolmogorov–Smirnov test. For normally distributed variables, statistical comparisons between groups were made using the independent samples *t*-test, one-way analysis of variance (ANOVA) with the Dunnett *t*-test, and associations were analyzed using the Pearson correlation analysis. For parameters not normally distributed, statistical comparisons between groups were made using the Mann–Whitney *U* tests, and associations were analyzed using the Spearman correlation analysis. The categorical variables were compared using the chi-square test. Stepwise multiple regression analysis was performed to determine independent factors associated with SFCT. The intraclass correlation coefficient (ICC) was estimated to assess the intraexaminer repeatability of the choroidal thickness measurements. *P* < 0.05 was considered statistically significant.

## 3. Results

### 3.1. The General Characteristics of Participants

Among the 120 children enrolled in the study, 59 (49.2%) were boys, and 61 (50.8%) were girls. The age of the children ranged from 8 years to 11 years old, with a mean age of 9.69 ± 1.12 years. Mean age of the boys and girls was 9.62 ± 1.17 years and 9.78 ± 1.05 years, respectively. The spherical equivalent refraction of the children ranged from −0.50 D to −5.75 D, with a mean of −2.88 ± 1.46 D. The axial length ranged from 22.67 to 26.20 mm, with a mean of 24.54 ± 0.79 mm.

### 3.2. Choroidal Measurements

The ICC for measurements of choroidal thickness was 0.976 (95% confidence interval, 0.940∼0.990, *P* < 0.001), indicating high repeatability of measurements of choroidal thickness. The SFCT of the children ranged from 108 to 359 *µ*m, with a mean of 252.80 ±46.95 *µ*m. The SFCT of boys and girls was 247.11 ±50.78 *µ*m and 253.95 ± 47.48 *µ*m, respectively. There was no significant difference in SFCT between boys and girls (*t*=0.758, *P*=0.45).

The mild myopia group consisted of 66 eyes. The moderate myopia group consisted of 54 eyes. Spherical equivalent was −1.72 ± 0.58 D in the mild myopia group and −4.31 D ± 0.77 D in the moderate myopia group. Axial length was 24.18  ± 0.69 mm (range, 22.67∼25.59 mm) in the mild myopia group and 24.97 ± 0.68 mm (range, 22.82∼26.20 mm) in the moderate myopia group. Subfoveal choroidal thickness was 262.00 ± 40.57 *µ*m (range, 195∼352 *µ*m) in the mild myopia group and 236.00 ± 55.08 *µ*m (range, 108∼359 *µ*m) in the moderate myopia group. The characteristics of the whole population and mild and moderate myopia groups are listed in Table 1. Mild myopia group had significantly higher spherical equivalent (*P* < 0.001), shorter axial length (*P* < 0.001), and thicker subfoveal choroidal thickness (*P*=0.005) than the moderate myopia group. No other differences between the two refraction groups were observed.

### 3.3. The Choroidal Topographic Variation in the Two Refraction Groups

**Figure 2:**
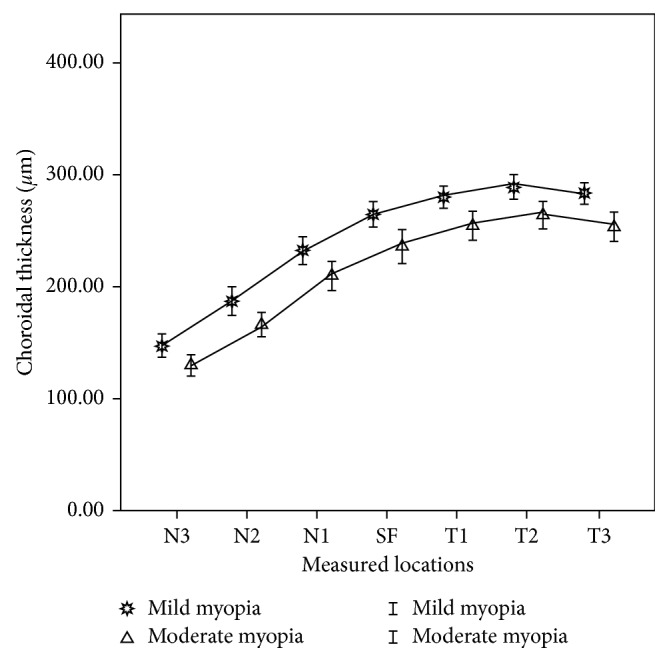
Choroidal thickness of horizontal measured locations in mild and moderate myopia groups: N1, N2, and N3: 1, 2, and 3 mm nasal to the fovea; SF: subfoveal; T1, T2, and T3: 1, 2, and 3 mm temporal to the fovea.

**Figure 3:**
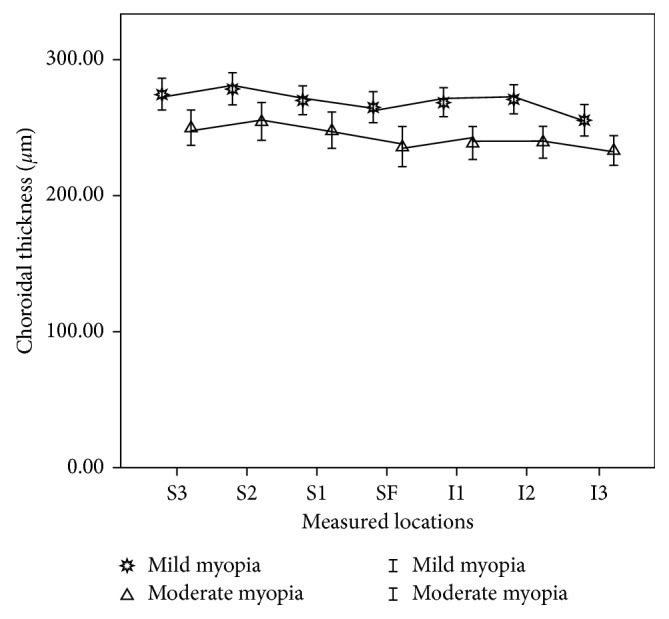
Choroidal thickness of vertical measured locations in mild and moderate myopia groups: S1, S2, and S3: 1, 2, and 3 mm superior to the fovea; SF: subfoveal; I1, I2, and I3: 1, 2, and 3 mm inferior to the fovea.

There were significant differences among the CTs of various locations in mild and moderate myopic groups (ANOVA, *F*=64.580, *P* < 0.001; *F*=38.892, *P* < 0.001, resp.). CTs of the mild myopia group were thicker than that of the moderate myopia group at all the measured locations. But the topographical changes among all the measured locations were similar between the two refraction groups. CTs of the measured locations nasal to the fovea were significant thinner than SFCT. The farther the location was away from the fovea the thinner the CT was. CTs thickened gradually in the other three directions and reached the maximum value at 2 mm to the fovea. But only the CT 2 mm temporal to the fovea was significantly thicker than SFCT. CTs became thinner at 3 mm to the fovea, but only the CT 3 mm inferior to the fovea was thinner than SFCT and without statistical significance. CTs of different measured locations and the significance of differences between SFCT and CTs of other measured locations are listed in Table 2. Figures 2 and 3 show the variation tendency of CTs of the two refraction groups.

### 3.4. The Relationships between Subfoveal Choroid Thickness and Age, Gender, Spherical Equivalent, and Axial Length

Associations of SFCT with age, gender, SE, and AL in the two refraction groups and in the whole population were investigated by correlation analysis (Table 3). There were significant negative correlations between AL and SFCT in the mild myopia group and the whole population. SE exhibited a significant positive association with SFCT in the whole population. There were no other significant correlations between SFCT and other parameters.

**Figure 4:**
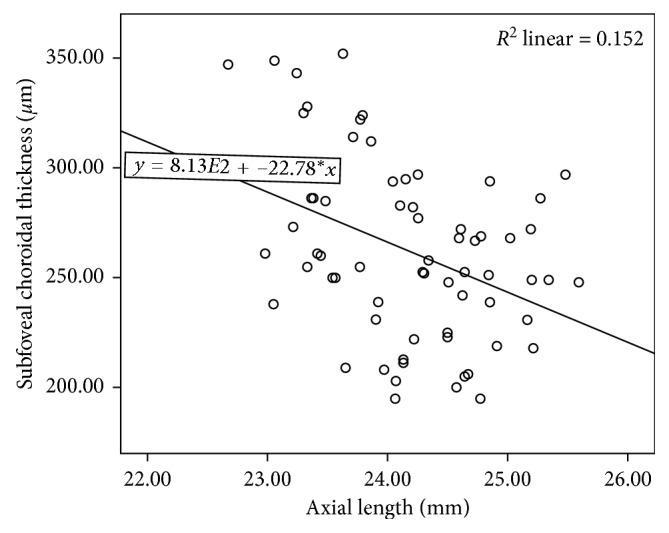
Association between subfoveal choroidal thickness and axial length in the mild myopia group.

**Figure 5:**
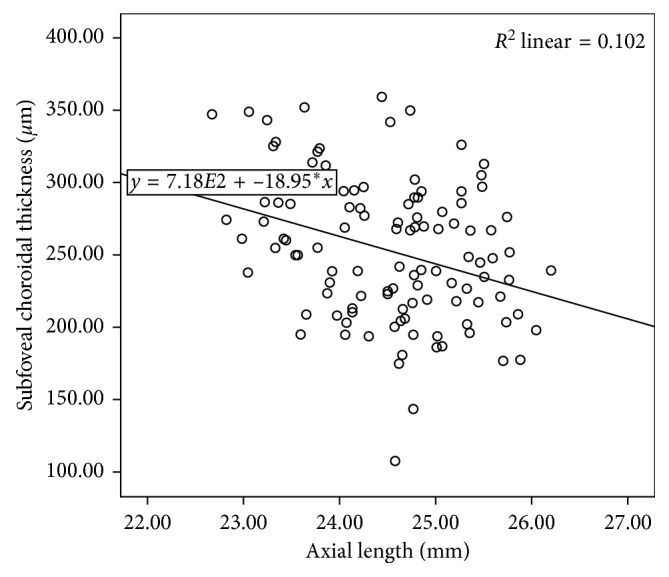
Association between subfoveal choroidal thickness and axial length in the whole participants.

Stepwise multiple regression analysis suggested that only axial length was the significant predictor of SFCT in the mild myopia group and in the whole participants. No variables were entered into the equation in the moderate myopia group. SFCT decreased by 22.78 *µ*m (*P*=0.001) for every 1 mm increase in axial length in the mild myopia group and 18.95 *µ*m (*P* < 0.001) in all. The predictive model was SFCT = 813 − 22.78 × AL (*F*=11.442, *P*=0.001, and  *R*^2^=0.152; Figure 4) in the mild myopia group and SFCT = 718−18.95 × AL (*F*=13.390, *P* < 0.001, and  *R*^2^=0.102; Figure 5) in the whole participant.

## 4. Discussion

### 4.1. Subfoveal Choroidal Thickness

Since Spaide et al. [18] firstly reported in vivo choroidal imaging with spectral-domain OCT and measured choroidal thickness, more and more researches have been conducted to study choroidal thickness in healthy population. However, the children's SFCT has been reported ranging from 260.4 ± 57.2 *μ*m to 359 ± 77 *µ*m [8, 9, 19–22]. The discrepancies can be partly explained by ethnicity differences. The different age (the narrowest range, 10∼15 years; the widest range, 3∼17 years) and refractive error (the narrowest range, +1.25 to −0.50 D; the widest range, +6 to −6 D) of the participants are among other factors that influence the data obtained in studies. To the best of our knowledge, this is the research of choroidal thickness of Chinese children with the narrowest age range. And choroidal thickness has been evaluated separately depending on different refraction groups. The SFCT of Chinese children aged from 8 to 11 years was 262.00 ± 40.57 *µ*m in the mild myopia group and 236.00 ± 55.08 *µ*m in the moderate myopia group. The mean SFCT of Chinese children had been reported ranging from 245 ± 66 *μ*m to 302 ± 63 *μ*m depending on different age and refractive error range [10–12, 23]. The SFCT of the mild myopia group in this research was consistent with a previous study of Chinese children, but SFCT of the moderate myopia group was thinner than that in the study [12]. Different age range might be the cause of different choroidal thickness.

The results might be inconsistent even in the studies of similar object. In the comparative studies of choroidal thickness between children and adults, the conclusions were different although the participants had similar age range (3∼15 years, 3∼17 years in the pediatric population versus 24∼87 years, and 25∼85 years in the adult population) [9, 19]. Nagasawa et al. [19] found that choroidal thickness of children was significantly thicker than that of adults. Ruiz-Moreno et al. [9] concluded that there were no significant differences of choroidal thickness between children and adults. The fluctuation of choroidal thickness due to large age range might contribute to the inconsistent results of the studies. During the period of growth and development, the thickness of choroid varies with age in children. One aim of this study was to obtain reliable reference values of choroidal thickness for specific pediatric population. The values based on narrow range of age and refractive error are more accurate and lead to more reliable and meaningful research conclusions.

### 4.2. Topographic Pattern of Choroidal Thickness

Margolis and Spaide [2] measured macular choroidal thickness in normal eyes at different points of horizontal section. They found that choroidal thickness varied topographically within the posterior pole. The choroid was thickest underneath the fovea and thinning rapidly in the nasal direction.

Comparative studies of distribution of macular choroidal thickness between high myopia and nonhigh myopia obtained similar results. The SFCTs were the greatest in nonhigh myopia groups but not in high myopia groups [6, 24, 25]. Sanchez-Cano et al. [26] investigated choroidal thicknesses of myopia and emmetropia. The choroid was thickest subfoveally in the emmetropia group but temporally and superiorly to the fovea in the myopia group. Zhang et al. [27] found that the choroidal thickness was greatest under fovea in low myopic eyes but in the temporal region in moderate and high myopic eyes.

But different findings were achieved in other researches. Tan et al. [28] studied the macular choroidal thickness of young adults whose refractive error range was −10.00 to + 0.50 D. The choroid was thickest temporal to the fovea inspite of the different refractive error. Harb et al. [29] measured the choroidal thickness of young adults whose refractive error was −5.3 ± 2.0 D (range, −13.1 to −0.9 D). The choroid was thickest subfoveal. Ruiz-Moreno et al. [9] compared macular choroidal thickness of children with that of adults. The choroid was thickest temporally to the foveal in children and subfoveally in adults. Other researches concerned of children population found that the thickest locations of choroid were not subfoveal both in myopic and nonmyopic children [8, 20]. In the studies of Chinese children, the thickest choroid were located similarly in myopic and nonmyopic participants, either temporal to the fovea [10, 11, 23] or subfoveal [12], even in the study of relatively large range of refractive error (−11.38 to +8.38 D) [10]. In this study, the topographic distributions of macular choroidal thickness in mild and moderate myopia children aged 8∼11 years were similar. The choroidal thickness thinned nasally and thickened gradually in the other three directions. Then, it thinned again after reaching the peak value at 2 mm to the fovea. The thickest choroid is located temporal to the fovea.

Lee et al. [30] suggested that the thickest point of macular choroid, which was originally subfoveal, might be “shifted” to the temporal side as the corresponding sclera and choroid had moved temporally during axial elongation. But the regulation was not the same in younger population [28, 29], especially in children. Macular choroid was not thickest at subfovea, either in myopic or in nonmyopic people [8–11, 20, 21, 23], and the thickest position did not change along with myopia progression. The similar topographical pattern of choroid thickness among different refractive statuses in children and young adults might be a stage of development. Choroid develops stable in adults at last. The choroidal thickness redistributes from children to adults, and the differences between nonmyopia and myopia, especially high myopia, are gradually significant.

To the best of our knowledge, this is the research of children macular choroidal thickness that had most and farthest measured locations in four directions. It enabled us to investigate the variation trend of choroidal thickness in the macular region thoroughly. The topographic distribution of macular choroidal thickness could not be detected if the measured locations were only horizontal [9], or not far from fovea enough [12], or only a few points [22].

### 4.3. Associations of Subfoveal Choroidal Thickness with Age, Gender, Spherical Equivalent, and Axial Length

In our study, the moderate myopia group had significant higher myopic refractive error, longer axial length, and thinner choroidal thickness than the mild myopia group. However, stepwise multiple regression analysis showed that only AL in the mild refractive group and in all subjects was significantly correlated with SFCT. This analysis predicted a decrease in SFCT of 22.78 *µ*m and 18.95 *µ*m for every 1 mm increase in axial length in the mild myopia group and all subjects. Correlation analyses revealed a significant positive association between SE and SFCT in the whole population (*r*=0.270, *P*=0.003), but the predictor was not significant in the final multiple regression model due to the colinearity between axial length and refractive error.

The association between AL and choroidal thickness differed between mild myopia and moderate myopia. AL and SFCT correlated significantly in the mild myopia group. But they had no significant correlation in the moderate myopia group. There are some reasons to be considered as follows. First, AL did not show a significant correlation with SFCT in the moderate myopia group because of narrow range of refractive error and variation in choroidal thickness between each individual. Second, sampling error was a possible cause. Third, maybe in different stages of myopia progression, the tendency of choroidal thickness to change with axial length differs. Mild myopia is the progressive stage of emmetropia to change to myopia, and the change of AL has a marked effect on SFCT. Moderate myopia is the stage that has not yet developed to high myopia after the onset of myopia. The thickness of choroid is relatively stable, and the correlation between AL and SFCT is not significant. Of course, this hypothesis needs to be verified by clinical researches with large sample size.

Associations of choroidal thickness with parameters of eyeball and body have been discussed in most papers [2–8, 10–12, 19–23, 25, 26, 29, 30]. Some of the conclusions were consistent and some of them contrary. SFCT correlated significantly with age, AL, and SE in most researches [2–7, 10, 11, 20–23, 25–27, 29], but they had no significant correlations in some studies [8, 12, 30]. In this study, SFCT correlated significantly with AL in the mild myopia group and all participants, and no other correlations had been found. The relatively narrow range of age and refractive error may be the possible causes. One of the purposes of this research was to investigate if the correlations between choroidal thickness and other parameters in narrow range of age and refractive error were similar to that of other studies. SFCT was relatively stable in narrow range of studies, as in [6, 8, 12], which had been mentioned in Introduction.

SFCT were found correlated negatively with age in participants older than 60 years and had no correlation with participants younger than 60 years [4]. There were also opposite findings [5]. In studies concerning children, some found that SFCT correlated positively with age [20–22], some found that SFCT correlated negatively with age [8, 19, 23], and some found that there were no correlations between them [12]. Only cross-sectional studies were not enough to outline the trend of choroidal thickness with age. Long-term longitudinal researches are needed to enrich the study of choroidal thickness and make the understanding of the choroid more comprehensive.

There were some limitations in this study. First, the measurements of choroidal thickness were performed manually and by only one examiner. Though the ICC in this study indicated high repeatability of choroidal thickness measurements, measuring error cannot be avoided. Automatic measurement software will be required for a more objective and efficient evaluation of choroidal thickness. Second, only 120 children enrolled in this study cannot represent all the children of this age, and the refractive status only included mild and moderate myopia. Others such as emmetropia and high myopia were not included in. This made the investigation of choroidal thickness in children not comprehensive enough. We designed this clinical research for most children beginning to have myopia after the age of 8, and mild and moderate myopia became common in children aged 8 to 11 years. Further researches of larger sample size and cover broader refractive status are needed to make the research more perfect. Third, longitudinal investigations should be conducted for cross-sectional studies which could not exhibit the tendencies of changes of choroidal thickness along with changes of age and refractive status in children.

## 5. Conclusions

This is a study of children choroidal thickness within a narrow range of age and refractive error. This study has the most and farthest measured locations of macular choroidal thickness in four directions. The topographic distributions of macular choroidal thickness in mild and moderate myopia were similar. The thickest choroid is located temporal to the fovea. AL in the mild refractive group and in all subjects was significantly negatively correlated with SFCT according to stepwise multiple regression analysis. No other significant correlations were found between SFCT and age, gender, and SE. SFCT was relatively stable in children in narrow range of age and refractive error.

## Figures and Tables

**Table 1:** General characteristics of the 120 participants and comparison between different refraction groups.

	Total (*n*=120)	Range	Mild myopia (*n*=66)	Range	Moderate myopia (*n*=54)	Range	*P*
Gender (M/F)	59/61	—	29/37	—	30/24	—	0.205^a^
Age (years)	9.69 ± 1.12	8 to 11	9.56 ± 1.08	8.17 to 11.75	9.85 ± 1.14	8.00 to 11.75	0.168^b^
SE (diopters)	−2.88 ± 1.46	−0.50 to −5.75	−1.72 ± 0.58	−2.75 to −0.50	−4.31 D ± 0.77	−5.75 to −3.00	<0.001^b^
AL (mm)	24.54 ± 0.79	22.67 to 26.20	24.18 ± 0.69	22.67 to 25.59	24.97 ± 0.68	22.82 to 26.20	<0.001^c^
SFCT (*µ*m)	252.80 ± 46.95	108 to 359	262.00 ± 40.57	195 to 352	236.00 ± 55.08	108 to 359	0.005^c^

M = male; F = female; SE = spherical equivalent; AL = axial length; SFCT = subfoveal choroidal thickness. ^a^Statistical significance was tested using the chi-square test. ^b^Statistical significance was tested using the Mann–Whitney *U* test. ^c^Statistical significance was tested using the independent samples *t*-test.

**Table 2:** Choroidal thickness of different measured locations and comparison between choroidal thickness of different measured locations and subfoveal choroidal thickness in two refraction groups.

Location	Mild myopia		Moderate myopia	
	CT (*µ*m)	*P* ^a^	CT (*µ*m)	*P* ^a^
SF	262.00 ± 40.57	—	236.00 ± 55.08	—
1 mm nasal	229.73 ± 44.35	<0.001	209.57 ± 48.46	0.028
2 mm nasal	183.24 ± 46.01	<0.001	166.15 ± 40.23	<0.001
3 mm nasal	147.12 ± 37.70	<0.001	129.37 ± 35.25	<0.001
1 mm temporal	276.47 ± 35.98	0.292	254.19 ± 47.40	0.272
2 mm temporal	284.68 ± 39.85	0.016	264.06 ± 45.54	0.016
3 mm temporal	279.23 ± 36.50	0.131	253.69 ± 47.26	0.303
1 mm superior	265.20 ± 39.41	1.000	247.72 ± 49.93	0.782
2 mm superior	273.36 ± 43.74	0.586	254.41 ± 51.71	0.259
3 mm superior	269.79 ± 44.67	0.921	249.54 ± 48.00	0.628
1 mm inferior	265.06 ± 40.03	1.000	238.46 ± 44.21	1.000
2 mm inferior	268.09 ± 40.87	0.985	239.20 ± 43.11	1.000
3 mm inferior	253.86 ± 46.24	0.898	232.89 ± 39.87	1.000

CT =  choroidal thickness; SF = subfoveal. ^a^Dunnett *t*-test.

**Table 3:** Correlations of subfoveal choroidal thickness with age, gender, spherical equivalent, and axial length.

	Total		Mild myopia		Moderate myopia	
	*r*	*P*	*r*	*P*	*r*	*P*
Gender	0.063	0.493^a^	0.064	0.609^a^	0.055	0.701^a^
Age	0.005	0.954^a^	0.151	0.226^a^	−0.116	0.417^a^
SE	0.270	0.003^a^	0.143	0.253^a^	0.225	0.112^a^
AL	−0.282	0.002^a^	−0.389	0.001^b^	−0.148	0.300^b^

SE = spherical equivalent; AL = axial length. ^a^Spearman correlation analysis. ^b^Pearson correlation analysis.
